# Chloral in Puerperal Eclampsia

**Published:** 1879-03-20

**Authors:** M. W. Warfield


### *CHLORAL IN PUERPERAL ECLAMPSIA.*

BY M. W. WARFIELD, M.D.

Mrs. E——, a primipara, æt. 35 years, is a tall, stout woman, of brunette complexion; married thirteen years. She has suffered from nausea and vomiting during the whole period of gestation. Four weeks since, I analyzed the urine, suspecting albuminuria, but obtained only negative results. I had made no examination since that time, nor had I seen her since, until her labor, January 7, when I found the patient still vomiting, and complaining of headache, although she had bled profusely from the nose. Face swollen and purple, eyelids puffy, hands stiff.

The os was dilated to the size of a half-dollar, rigid. Head presenting in first position. The pains were regular.

The case progressed without accident. Bowels were moved by an active cathartic. Kidneys seemed sufficiently active until the bearing-down pains came on. At two o'clock P.M. Mrs. E. was suddenly seized with a violent convulsion. With such delay only as was caused by a lancet rusty from long disuse, I relieved the patient of a quart of blood (without arresting the convulsions), and sent to my office, three-quarters of a mile distant, for chloral hydrate and chloroform. When the messenger returned, as the patient was enable to swallow, I administered, per rectum, forty-five grains of chloral hydrate, using chloroform (of which I had but little) on a handkerchief until she came fully under the influence of the chloral. The spasms were arrested. I repeated the chloral injections as often as the effect seemed to wear off, until the close of the labor, which occurred about four o'clock P.M., when Mrs. E. was delivered of a female child weighing nearly ten pounds. I left my patient about seven P.M., restored to consciousness and able to converse, but was startled by a messenger, two hours later, who reported Mrs. E. to be dying. I hastened to the house, and found my patient quite delirious and very restless, requiring some one to hold her in the bed. She had had a strong convulsion before my arrival. Again administered the chloral hydrate, this time ninety grains to the dose, with the same result as before. Stayed by the patient all night, and renewed the chloral as required, adding laudanum when my chloral was getting exhausted. Just before day, I was aroused from a doze, to hear her talking rationally and inquiring what had happened, etc.

As soon as Mrs. E. could swallow, I put her upon the following prescription, although I was not sure that her condition was due to uræmia:

℞ Bitartrate potass.................... ℥ iss.

Fl. ext. digitalis.................... ʒ ss.

Syrup. squill........................ ℥ ss.

Swt. spts. nitre..................... ℥ ii.

Water to make ....................... ℥ viii.

Dose, two tablespoonsful every two hours.

To relieve the headache and guard against any return of the spasms, I ordered between these doses twenty grains bromide potass., all of which has acted favorably, and my patient is now convalescing rapidly.

My object in reporting this case is to impress on the profession the value of chloral in the treatment of eclampsia. In the course of my parctice I have had a number of such cases, in only one of which (occurring about the fourth month of pregnancy) was I able to arrest convulsions until delivery had been accomplished, although chloroform, bleeding, etc., had been tried. I attribute my success in the above case entirely to the free use of chloral hydrate.—*Med. Times.*

